# The behavioral and neural effects of parietal theta burst stimulation on the grasp network are stronger during a grasping task than at rest

**DOI:** 10.3389/fnins.2023.1198222

**Published:** 2023-10-26

**Authors:** Elana R. Goldenkoff, Joseph A. Deluisi, Danielle P. Destiny, Taraz G. Lee, Katherine J. Michon, James A. Brissenden, Stephan F. Taylor, Thad A. Polk, Michael Vesia

**Affiliations:** ^1^School of Kinesiology, University of Michigan, Ann Arbor, MI, United States; ^2^Department of Psychology, University of Michigan, Ann Arbor, MI, United States; ^3^Department of Psychiatry, University of Michigan, Ann Arbor, MI, United States

**Keywords:** transcranial magnetic stimulation, state-dependency, manual dexterity, functional connectivity, plasticity, theta burst stimulation, posterior parietal cortex, motor cortex

## Abstract

Repetitive transcranial magnetic stimulation (TMS) is widely used in neuroscience and clinical settings to modulate human cortical activity. The effects of TMS on neural activity depend on the excitability of specific neural populations at the time of stimulation. Accordingly, the brain state at the time of stimulation may influence the persistent effects of repetitive TMS on distal brain activity and associated behaviors. We applied intermittent theta burst stimulation (iTBS) to a region in the posterior parietal cortex (PPC) associated with grasp control to evaluate the interaction between stimulation and brain state. Across two experiments, we demonstrate the immediate responses of motor cortex activity and motor performance to state-dependent parietal stimulation. We randomly assigned 72 healthy adult participants to one of three TMS intervention groups, followed by electrophysiological measures with TMS and behavioral measures. Participants in the first group received iTBS to PPC while performing a grasping task concurrently. Participants in the second group received iTBS to PPC while in a task-free, resting state. A third group of participants received iTBS to a parietal region outside the cortical grasping network while performing a grasping task concurrently. We compared changes in motor cortical excitability and motor performance in the three stimulation groups within an hour of each intervention. We found that parietal stimulation during a behavioral manipulation that activates the cortical grasping network increased downstream motor cortical excitability and improved motor performance relative to stimulation during rest. We conclude that constraining the brain state with a behavioral task during brain stimulation has the potential to optimize plasticity induction in cortical circuit mechanisms that mediate movement processes.

## Highlights

- Controlling the brain state during TMS with a grasping task improves motor performance.
- Brain-state-dependent parietal TMS induces immediate changes in the motor cortex.
- Brain-state-dependent TMS can enhance the impact of neuromodulation on motor function.

## Introduction

Goal-directed hand actions, such as grasping for objects, are integral to human behavior. Performing such behaviors activates a widespread network of cortical areas, including the prefrontal cortex, premotor cortex, and posterior parietal cortex (PPC; Grafton, 2010; Davare et al., 2011; Turella and Lingnau, 2014; Fattori et al., 2015; Gallivan and Culham, 2015). The primary motor cortex (M1) plays an essential role in motor control and is part of a more extensive parietal–frontal network involved in many aspects of movement planning and decision-making (Kalaska et al., 1997; Andersen and Cui, 2009; Cisek and Kalaska, 2010; Crawford et al., 2011; Vesia and Crawford, 2012). Neural inputs from PPC to motor areas in the frontal lobe are generally thought to mediate motor commands for hand movements (Grafton, 2010; Turella and Lingnau, 2014; Fattori et al., 2015; Gallivan et al., 2018). Current evidence from functional cortico-cortical connectivity measures derived from dual-site transcranial magnetic stimulation (dsTMS) indicates that inputs from PPC exert a facilitatory influence on motor output during the preparation and execution of hand-movement planning, suggesting a functional parietal-motor connection that controls hand muscles (Koch et al., 2007, 2008, 2010; Koch and Rothwell, 2009; Davare et al., 2010; Ziluk et al., 2010; Vesia and Davare, 2011; Karabanov et al., 2013; Vesia et al., 2013, 2017; Koch, 2020). The plasticity of M1 associated with voluntary movements and motor-skill learning also appears to be influenced by distributed activity in functionally related brain areas in the motor network (Sanes and Donoghue, 2000; Hardwick et al., 2013; Buch et al., 2017). However, it is unclear if other brain areas, such as PPC, can modulate this motor plasticity.

Repetitive transcranial magnetic stimulation (rTMS) can induce plastic changes in the brain (Hallett, 2007). For instance, intermittent theta burst stimulation (iTBS), a form of rTMS, can produce durable increases in motor cortical excitability for a period that outlasts the stimulation when applied to M1 (Huang et al., 2005; Suppa et al., 2016). The mechanisms of these changes are caused by processes analogous to long-term potentiation (LTP) that are also seen with skill learning (Capocchi et al., 1992; Bear and Malenka, 1994; Berardelli et al., 1998). This stimulation can directly modify neural activity at the locus of stimulation, as well as the activity of interconnected and functionally coupled brain areas (Siebner et al., 2009b). These persistent effects on neural activity are primarily thought to be constrained within the functional network of the targeted region (Fox et al., 2012). Therefore, it is unsurprising that rTMS can be particularly effective for treatment-induced behavior improvements when applied to functional brain networks (Raffin and Siebner, 2014; Silasi and Murphy, 2014; Fox, 2018; Horn and Fox, 2020). Yet, the persisting effects of rTMS on enduring motor cortical excitability and behavioral outcomes are highly variable and poorly understood (Ziemann and Siebner, 2015).

The variability of rTMS-induced effects on brain and behavior responses can be partly explained by variations in ongoing activity levels of functionally specific neural populations and pathways at the time of stimulation (Silvanto et al., 2008; Romei et al., 2016; Bergmann, 2018). For example, recordings in the visual cortex indicate that the post-stimulation response depends on pre-stimulation activity levels (Pasley et al., 2009). Similarly, pairing rTMS with visual stimuli has shown a direction-selective plasticity induction in the visual system that biases subsequent behavioral responses for a particular motion direction (Chiappini et al., 2018). Therefore, the functional context of neural activity during stimulation appears necessary for targeting brain networks associated with specific functions.

Although we and others have shown that the PPC and associated parietal–frontal circuits of the motor planning network are essential for skilled grasp control (Davare et al., 2010, 2011; Grafton, 2010; Vesia and Crawford, 2012; Turella and Lingnau, 2014; Fattori et al., 2015; Gallivan and Culham, 2015; Vesia et al., 2017), the notion that the functional context of brain activity during PPC stimulation can modulate interactions with functionally connected motor regions to alter plasticity associated with motor control has not been directly tested. We utilized a novel approach that combines an object-driven grasp task, which selectively activates the motor control network, with iTBS to PPC. In the pilot study (Experiment 1), we investigated the immediate effects of state-dependent stimulation on electrophysiological and behavioral responses. Experiment 2 replicated the findings from the pilot study with a larger sample size and additional stimulation sessions. We predicted that applying parietal iTBS while constraining the brain state via a grasping task will be more likely to increase motor cortical excitability than an application of the same stimulation protocol during rest. We also predicted that motor performance improvement would be greater after parietal iTBS during grasp performance compared to parietal iTBS at rest.

## Materials and methods

### Participants

We conducted two experiments involving 72 healthy, right-handed participants (Oldfield, 1971). In Experiment 1 (pilot study), we studied 24 adult participants (13 females and 11 males aged between 18 and 30). For our second experiment, we recruited 48 participants (32 females and 16 males, 18–50 years) and assigned 16 participants to each group. The sample size was determined based on prior research (Fiori et al., 2018), considering a motor performance effect size of 0.11, a desired power of 0.8, a significance level (α) of 0.05, and an estimated dropout rate of 10%. All participants provided written informed consent and underwent a TMS Adult Safety Screen to assess the potential risk of adverse reactions to TMS (Keel et al., 2001; Rossi et al., 2011). The Institutional Review Board at the University of Michigan (IRB#: HUM00157197 and HUM00186637) approved experimental procedures in accordance with the Declaration of Helsinki.

### Electromyographic recordings

Electromyography (EMG) activity of the right hand was recorded from the first dorsal interosseous and abductor pollicis brevis muscles using surface electrodes (Ag-AgCl, 9-mm diameter). The active electrode was placed over the muscle belly, and the reference electrode over the metacarpophalangeal joint of the finger. Signals were amplified (×1000), band pass filtered (20 Hz–2.5 kHz; Intronix Technologies Corporation, Model 2024F), digitized at 5 kHz using a Micro 1,401 data acquisition interface controlled by Signal Software version 7 (Cambridge Electronic Design Ltd.), and stored on a computer for off-line analysis.

### Transcranial magnetic stimulation

Monophasic pulses were delivered from two separate Magstim model 200^2^ stimulators (Magstim) through a D70^2^ (loop diameter, 70 mm) or D50 Alpha B.I. (loop diameter, 50 mm) figure-8 coil. First, motor-evoked potentials (MEPs) in the targeted relaxed right-hand muscle were elicited by delivering single-pulse TMS (spTMS) over the hand area of the left primary motor cortex (M1). The TMS coil was placed tangential to the scalp and at a 45° angle from the midsagittal line. The placement of the TMS coil was adjusted to the location where TMS produced the largest MEP from the targeted right-hand muscles. Next, the TMS coil’s position was marked and registered using a standard MRI template with a frameless stereotactic neuronavigation system (Brainsight 2, Rogue Research Inc.). The resting motor threshold (RMT) was determined by the minimum stimulator output needed to obtain MEP amplitudes of at least 50 μV in five of ten TMS pulses with the D50 Alpha B.I. coil when the muscle was completely relaxed (Rossini et al., 1994, 2015; Groppa et al., 2012). The intensity of the D70^2^ coil was adjusted to induce MEP amplitudes of about 1 mV in at least five out of ten trials in the relaxed targeted right-hand muscle.

### Stimulation target identification

**Figure 1:**
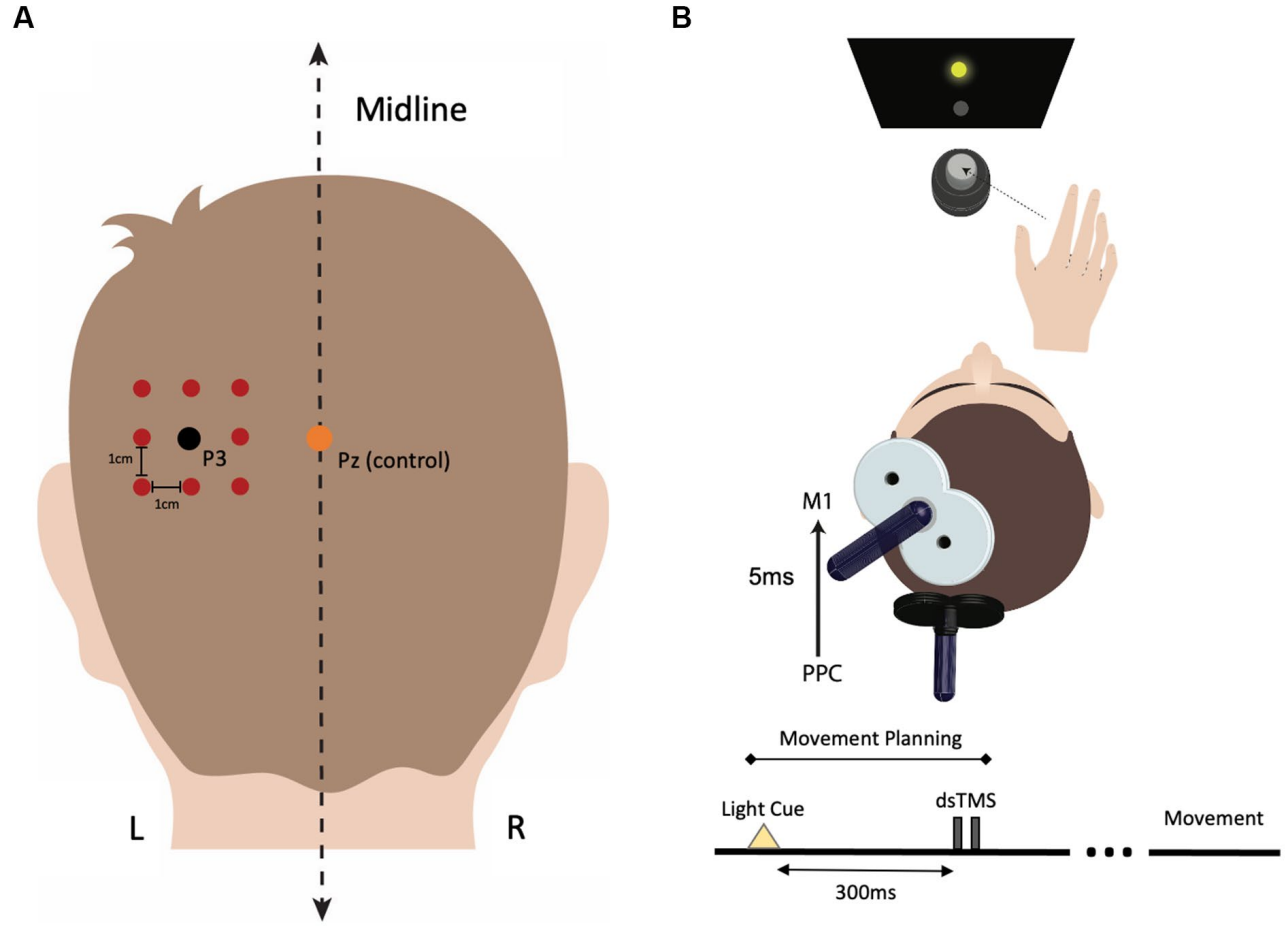
Procedure for identifying individualized left parietal stimulation locations. **(A)** The P3 electrode location was marked for each participant using the 10–20 EEG system. A 3 × 3 square search grid, with each point separated by 1 cm, was centered around P3 using Brainsight stereotactic software. The Pz electrode position was used as the stimulation location for the control condition. **(B)** During the functional localization protocol, each grid location over the parietal cortex was assessed for its maximum facilitatory effect on the primary motor cortex (M1). To identify the participant-specific location in PPC where stimulation induced the greatest facilitation in motor-evoked potential (MEP) amplitude, a dual-site, paired-pulse TMS (dsTMS) paradigm was employed using two coils. During dsTMS, participants performed an object-directed grasp task, and dsTMS was applied 300 ms after the movement cue (LED flash) occurred, coinciding with the planning phase of movement. Electromyography (EMG) was used to measure changes in MEP amplitude during the planning phase of the movement. The conditioning pulse intensity over PPC was 90% of the resting motor threshold (RMT), and the test pulse intensity over M1 was adjusted to induce an MEP of ~1 mV in the target hand muscle. The interstimulus interval (ISI) between the conditioning and test pulse was set at 5 ms. Approximately ten dsTMS pairs were delivered at each search grid location, and the location that induced the largest MEP response in three of five consecutive trials was selected as the PPC rTMS location.

**Figure 2:**
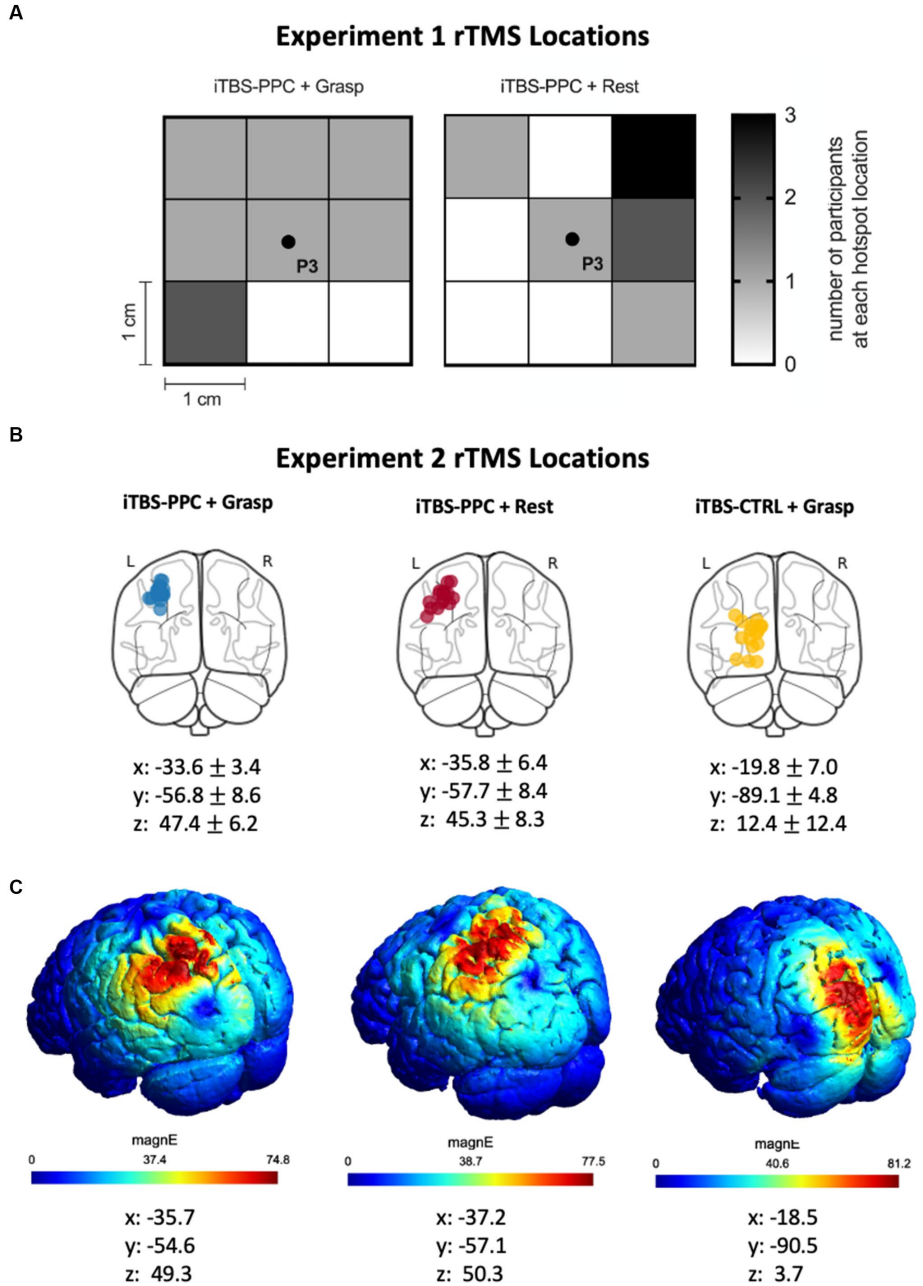
**(A)** Heat map indicating the search grid spot that was selected for the participants in the iTBS-PPC + Grasp and iTBS-PPC + Rest groups in Experiment 1. **(B)** In Experiment 2, structural fMRI was used to identify individualized left parietal stimulation locations for both iTBS-PPC + Grasp and iTBS-PPC + Rest groups. For the iTBS-CTRL + Grasp group, a non-correlated region was selected as the stimulation location. The stimulation locations for each participant are indicated on a standard brain, and MNI coordinates (mean ± SD) are shown for each group. **(C)** The electric field induced by transcranial magnetic stimulation (TMS) for one participant from each group is shown.

We used a function-based search-grid dsTMS technique to establish individualized left PPC locations for Experiment 1. This method uses a “hunting procedure” to target personalized functional interactions in the cortical grasping network. First, the left parietal stimulation target was selected as the P3 (left PPC) electrode position on the 10–20 electroencephalogram (EEG) coordinate system (Herwig et al., 2004; Okamoto et al., 2004) using commercially available 10–20 EEG stretch caps (g.GAMMAcap, g.tec Medical Engineering) in each participant. The P3 electrode location has been previously shown to target the inferior parietal lobule (Vesia et al., 2006, 2008, 2010, 2015). A square, 3 × 3 search grid, with positions separated by 1 cm, centered on the P3 target, was created using Brainsight (Figure 1A). A dsTMS approach with two coils was then used to identify participant-specific stimulation locations in the left PPC where parietal stimulation effectively exerts grasp-specific facilitation on M1 during an object-directed grasp task. This dsTMS technique provides a means for assessing how the behavioral context modulates the strength of interaction between PPC and M1 when the grasp-task demand recruits the parietal-motor circuit (Koch and Rothwell, 2009; Vesia and Davare, 2011; Vesia et al., 2013, 2017; Bestmann et al., 2015; Lafleur et al., 2016; Hallett et al., 2017; Goldenkoff et al., 2020). Specifically, we adopted a paradigm used previously by our group to activate the PPC-M1 circuit early in the motor plan for grasp movements (Vesia et al., 2013, 2017). Participants made one of two object-directed grasp movements to a target object with the right hand (Figure 1B). The target object was a small cylinder (2.5 cm diameter, 6.5 cm height) fixed atop a larger cylinder (7 cm diameter, 6.5 cm height), located 30 cm in front and 10 cm to the right of the starting hand position. Participants maintained visual fixation on two central LEDs in the midline for 2 s. Participants were instructed to grasp: (1) the top cylinder with a precision grip when the top LED flashed or (2) the bottom cylinder with a whole-hand grasp when the bottom LED flashed. To probe causal connectivity between the PPC and M1 in the left hemisphere, a conditioning stimulus (CS) over each PPC target in the grid was applied before delivering a test stimulus (TS) to ipsilateral M1 during reaction time (i.e., action plan phase) of the object-directed grasp such that the MEP recordings were collected before actual movement initiation (Vesia et al., 2017). TS intensity was adjusted to induce MEP amplitudes of about 1 mV. CS preceded TS by an interstimulus interval (ISI) of 5 ms at a stimulation intensity of 90% RMT (Koch et al., 2008; Vesia et al., 2013, 2017). Approximately ten TS and CS-TS were administered randomly at each grid position. The optimal scalp position for coil placement over the left PPC was defined as the point on the grid where CS elicited the largest MEP exceeding 1.2 mV from the contralateral hand muscle of the right (response) hand in three of five consecutive trials (Oliver et al., 2009; Karabanov et al., 2013). Brainsight was used to accurately place both coils throughout the localization of the parietal stimulation target. The stimulation location for the control condition was set at the Pz electrode position, which is not part of the parietal-motor circuit responsible for grasping. The parietal rTMS location on the grid was recorded and reported in Figure 2A.

In our second experiment, the site for PPC stimulation and the cortical control stimulation locations were identified using structural MRI data on each participant. MRI data were acquired using a 3 T GE scanner (MR 750) with a 32-channel head coil. T1-weighted structural images were obtained for anatomical localization. To locate the individualized left parietal stimulation target, we generated a region of interest mask based on the superior medial parietal regions ‘L_LIPv, L_7PC’ using the MNI projection of the HCP-MMP1 atlas (Glasser et al., 2016). We chose a point within the anatomical mask that overlapped the center of a gyrus. We identified a target in the left visual cortex for the control stimulation group based on anatomical criteria. Notably, the visual cortical region is outside the grasping network. We determined the cortical location reached by the stimulation in each participant by projecting the coil location on the scalp onto their individual MRI using Brainsight. The resulting coordinates were reported in MNI space (Figure 2B). To visualize the data, SimNIBS 4.0 was used to estimate the TMS-induced electric fields (Thielscher et al., 2015; Figure 2C).

### Theta burst stimulation

Intermittent theta burst stimulation (iTBS) to the left cortical targets was administered using a MagPro X100 with MagOption (MagVenture Inc.) and a statically cooled figure-8 coil (MCF-B70). iTBS consisted of three pulses at a frequency of 50 Hz every 200 ms for 2 s and repeated every 10 s for a total of 190 s (600 pulses; Huang et al., 2005). The conventional approach for individualizing iTBS intensity is based on the motor threshold response, which uses an intensity of 80% of the active motor threshold (AMT; Huang et al., 2005). AMT was defined as the lowest intensity required for eliciting MEP of 200 μV in five of ten consecutive trials during a 20% maximum voluntary contraction of the muscle in the right hand with the MCF-B70 coil using biphasic pulses (Huang et al., 2005). We assessed AMT for each participant to compare our stimulation intensity with previous studies that utilized the conventional approach and ensure that our stimulation intensity adhered to safety guidelines (Oberman et al., 2011; Rossi et al., 2021).

The pilot study (Experiment 1) delivered iTBS at a fixed percentage of maximum stimulator output (% MSO) of 40% to decrease the inter-individual difference in stimulation-induced effects (Vesia et al., 2010). This methodological adjustment is based on evidence demonstrating that motor threshold does not adequately characterize the underlying physiology of non-motor areas of the brain (Stewart et al., 2001; Stokes et al., 2005; Khammash et al., 2020). For Experiment 2, we administered iTBS using a personalized stimulation intensity based on the individual participant’s functional neuroanatomy. This personalized approach adjusted AMT according to the distances between the scalp and the underlying cortex (for details, see Stokes et al., 2005). The stimulation intensity was then set at 80% of the adjusted AMT for each participant (iTBS_PPC + Grasp_: 37.4 ± 3.2; iTBS_PPC + Rest_: 36.7 ± 2.7; iTBS_Control + Grasp_: 37.4 ± 3.2).

### Assessment of motor cortical excitability

Long-term potentiation-like plasticity in M1 was assessed by quantifying the changes in the level of motor cortical excitability with the different stimulation protocols (Chen and Udupa, 2009). A fixed percentage of maximum stimulator output was used to elicit MEPs of about 1 mV peak-to-peak amplitude using spTMS with the D70^2^ coil before iTBS to PPC. Twenty-four MEPs were recorded at every assessment time point before and after (Experiment 1: 0, 15, 30, 45, and 60 min; Experiment 2: 30 and 60 min) each intervention with the 1 mV TMS intensity determined before each intervention. Stimuli were applied every 5 s.

### Assessment of motor performance

Motor performance was assessed by examining changes in the speed to complete a widely used nine-hole pegboard manual dexterity test. The pegboard task requires dexterous control of complex movements such as multi-digit grasping and manipulating small objects (Mathiowetz et al., 1985; Grice et al., 2003; Bunday and Perez, 2012; Fiori et al., 2018). Performance of the pegboard task engages parietal–frontal brain areas in the cortical grasping network subserving sensorimotor functions (Davare et al., 2011; Bunday and Perez, 2012; Fiori et al., 2018). Participants were seated in front of a table with the start position of the right hand positioned 10 cm from the pegboard apparatus. Behavioral performance on the pegboard task was evaluated by measuring the time to complete the task using a stopwatch every time before and after (30 and 60 min) the intervention.

A choice-reaction visuomotor task (CRT) was used as a control task to assess visuomotor function because it does not involve dexterous shaping and manipulating objects by the hand as required by the pegboard task (Fiori et al., 2018). Therefore, CRT is thought to be less associated with the PPC-to-M1 neural pathway. Participants were seated in front of a monitor and viewed stimuli (central number cue: ‘1’ or ‘2’) from 30 cm. Participants were instructed to respond by pressing the ‘1’ or ‘2’ key on the keyboard with the right index or middle finger. Participants were instructed to perform the task as quickly and as accurately as possible. Participants performed 40 trials at every time point before and after (30 and 60 min) the intervention. Visual stimuli were presented, and the mean reaction time (RT) of hand responses was recorded using PsychoPy (version 2021.2.3; Peirce et al., 2019). RT was defined as the interval between the visual number cue and the correct key button response. Before each experimental session, participants were familiarized with the pegboard and CRT tasks during a short training period with an instructional video^1^ followed by a practice block.

### Experimental design

**Figure 3:**
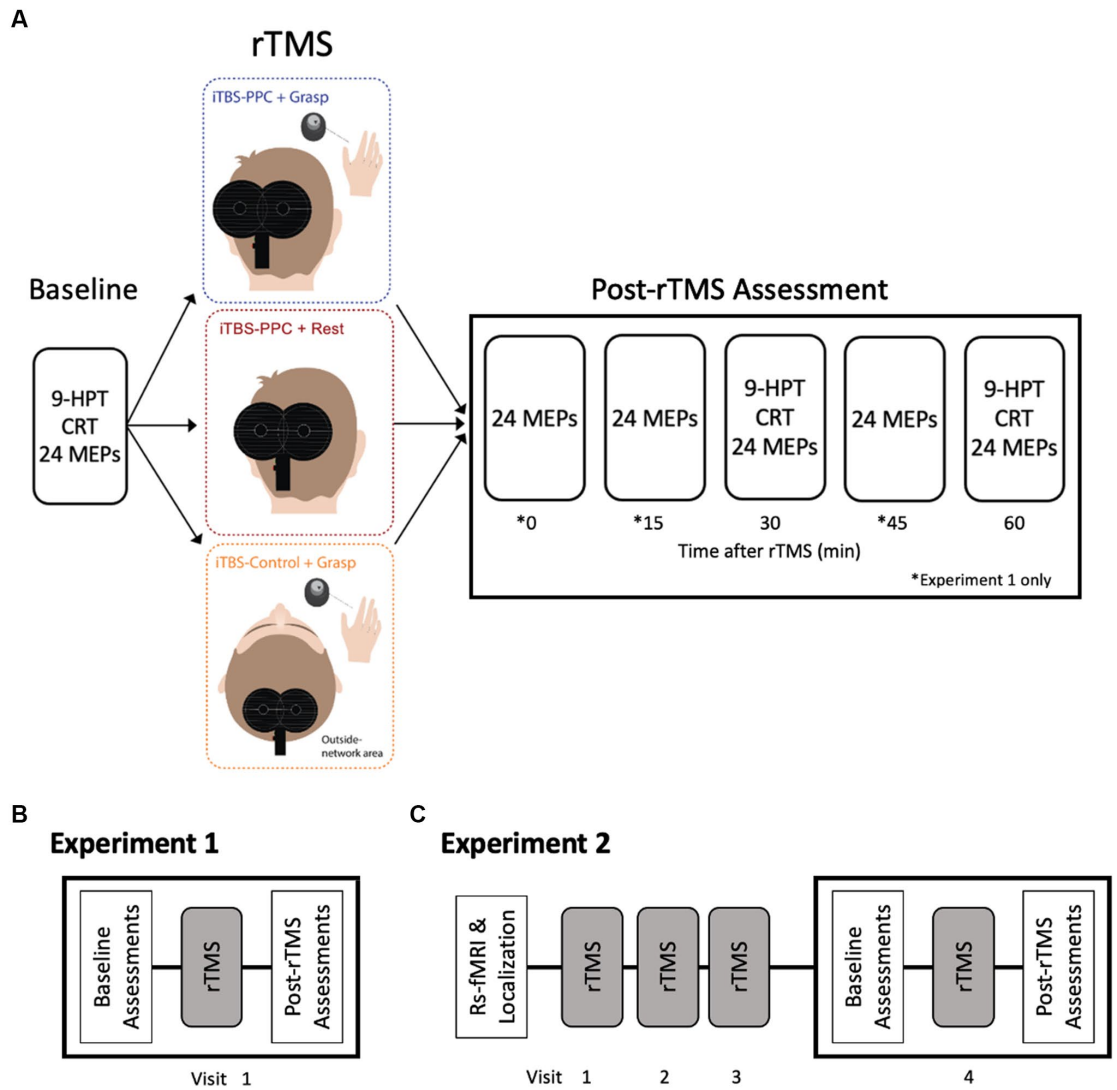
Experimental Design. **(A)** Participants were randomly assigned to one of three rTMS intervention groups. Electrophysiological and behavioral measurements were taken before (Baseline) and for an hour after the stimulation intervention. Motor-evoked potential amplitudes were measured at baseline and every 15 min for an hour after the iTBS intervention (0, 15, 30, 45, and 60 min) in Experiment 1 and after 30 and 60 min in Experiment 2. Both experiments measured behavioral performance on a nine-pegboard task (9-HPT) and choice reaction task (CRT) before and after the intervention (30 and 60 min). **(B)** In Experiment 1, participants underwent a single session of stimulation. **(C)** In Experiment 2, participants underwent a structural fMRI scan to determine the parietal stimulation location. Participants received three consecutive daily sessions of rTMS. On Visit 4, participants underwent assessments before (Baseline) and after rTMS, similar to Experiment 1.

Our study randomly assigned participants to one of three rTMS intervention groups, followed by electrophysiological measures with TMS and behavioral measures (Figure 3A). Participants in the first group received iTBS to the PPC while concurrently performing a grasping task (iTBS_PPC + Grasp_). Participants in the second group received iTBS to the PPC while in an unconstrained, resting state (iTBS_PPC + Rest_). Contrasting these groups allowed us to elucidate the effects of targeted TMS enhancement of parietal–frontal grasping network and motor function and the interaction between parietal iTBS and behavioral state. To test the functional specificity of stimulation to the PPC, a third group of participants received iTBS to a cortical region outside of the grasping network while concurrently performing a grasping task (iTBS_Control + Grasp_).

In Experiment 1 (pilot study), 24 participants received a single session of iTBS aimed at either the PPC or Pz electrode position (Figure 3B). In the second experiment, 48 participants underwent four consecutive daily sessions of iTBS, targeting either the PPC or the cortical control site. Each assessment session tested for the effects of each iTBS protocol on motor cortical excitability (e.g., MEPs) and behavioral performance (e.g., pegboard task and CRT). To measure changes in MEP amplitude, spTMS was applied to M1 at a fixed intensity that produced an MEP of 1 mV. In Experiment 1, we measured MEP amplitudes at baseline and every 15 min for an hour after the iTBS intervention (0, 15, 30, 45, and 60 min). In the second experiment, we measured MEP amplitudes immediately before and after (30 and 60 min) the fourth iTBS session (Figure 3C). Brainsight was used to place the D702 coil over M1 throughout the experiment accurately. Both experiments assessed manual dexterity and CRT before and after the iTBS intervention (30 and 60 min).

### Statistical analysis

Separate one-way analyses of variance (ANOVA) were used to confirm that the three groups (iTBS_PPC + Grasp.,_ iTBS_PPC + Rest_, iTBS_Control + Grasp_) did not differ in age or motor cortical excitability at baseline. MEP amplitudes were measured peak-to-peak for maximum and minimum values in the time window between 10 and 50 ms after spTMS (Carson et al., 2004; Fujiyama et al., 2016; Vesia et al., 2018). Changes in motor cortical excitability across Time and Intervention group were tested by fitting a linear mixed-effects model. The transformed MEP amplitude was used as the dependent variable, with the Intervention group and Time as fixed effects and subject as a random effect. Before including the data in the model, outlier MEP amplitudes that deviated by more than 3 units from the absolute median were removed for each subject. In total, 3.5% of all MEPs were excluded in Experiment 1, and 4.2% were excluded in Experiment 2 (Leys et al., 2013). MEP amplitudes were further transformed to account for their non-normal distribution. In Experiment 1, a power transformation of x^−0.16^ was used, while in Experiment 2, a power transformation of x^0.017^ was applied. The model was then tested using type II Wald F tests with Kenward–Roger degrees of freedom correction.

Changes in motor performance were quantified by expressing mean time as a symmetric percentage change from the baseline of the time to complete the pegboard task and mean reaction time on the CRT for each participant. For the pegboard task, symmetric percentages were subjected to an order norm transformation to meet normality assumptions. Then, a linear mixed-effects model was fitted with transformed symmetric percent change as the dependent variable, Intervention group (iTBS_PPC + Grasp.,_ iTBS_PPC + Rest_, iTBS_Control + Grasp_), and Time (30, 60 min) as fixed effects, and subject as a random effect. Similarly, for reaction time, values were log-transformed for normality. A linear mixed-effects model was fitted with transformed reaction time as the dependent variable, Intervention group and Time as fixed effects, and subject as a random effect. After fitting each model, type II Wald F tests with Kenward–Roger degrees of freedom correction were used to test for differences.

In addition, Games-Howell *post hoc t*-tests were performed on pairwise comparisons of groups to account for unequal variances between groups and control for multiple comparisons’ Type I error rate (Games and Howell, 1976). Analyses were performed using IBM SPSS Statistics Version 26.0 (IBM Corp., Armonk, NY, United States) and R (R Core Team, Vienna, Austria, 2022). Data are given as mean ± standard error of the mean (SEM). The threshold for statistical significance was set at *p* ≤ 0.05. Where appropriate, partial *η* squared (*η*_p_^2^) values were computed as a measure of effect size. Cutoffs for effect sizes of ≥0.01, ≥0.06, and ≥ 0.14 are considered small, medium, and large, respectively (Cohen, 1992).

## Results

**Figure 4:**
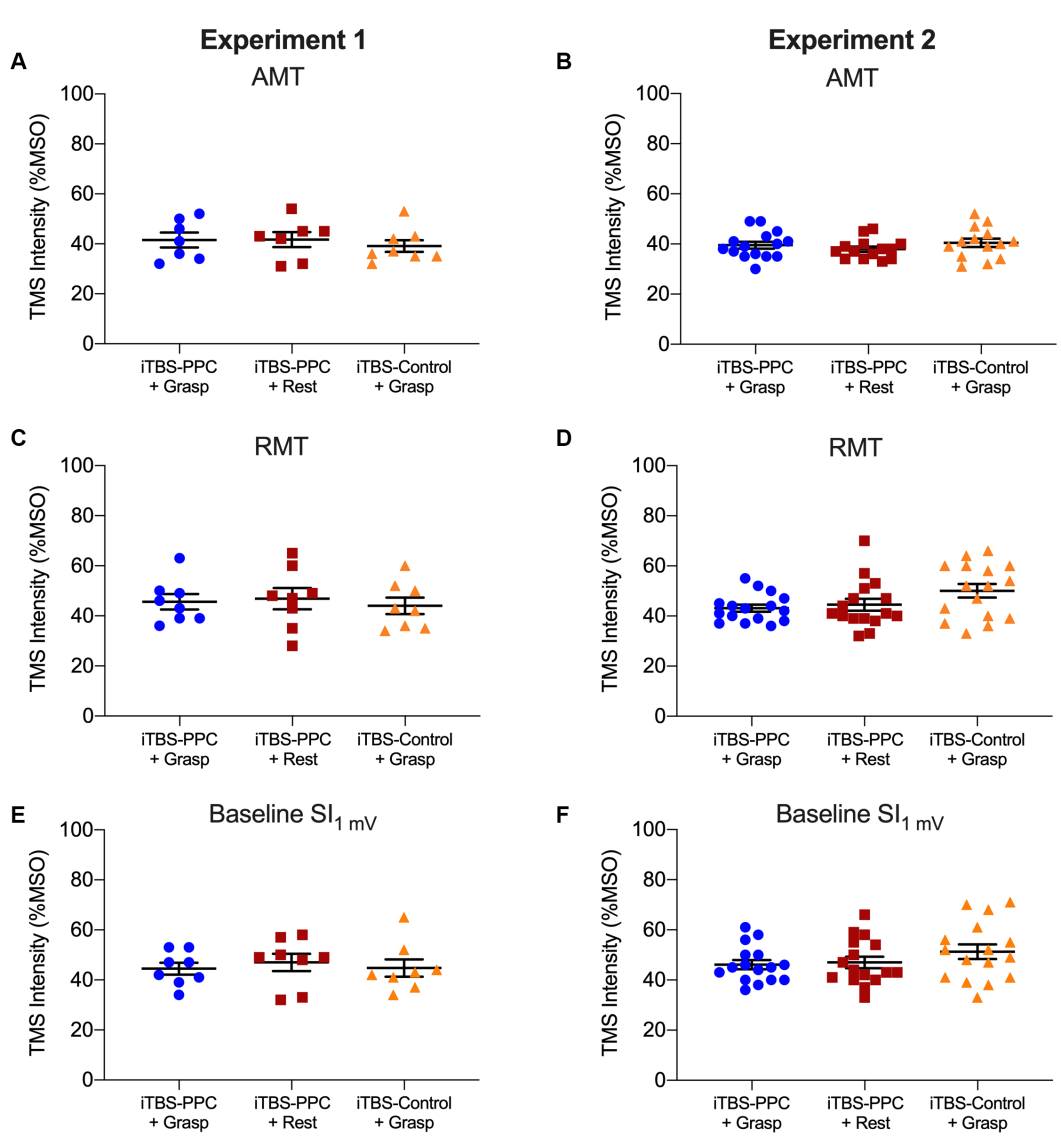
Column scatter plots showing the (A-B) transcranial magnetic stimulation (TMS) intensity (expressed as a percentage of the maximum stimulator output, MSO) of active motor threshold (AMT), (C-D) TMS intensity of resting motor threshold (RMT), and (E-F) TMS intensity eliciting 1 mV MEPs at baseline, for each participant for the iTBS_PPC + Grasp_ group (blue circles) and iTBS_PPC + Rest_ group (red squares) and iTBS_Control + Grasp_ group (orange triangles) for each experiment. No differences were found between groups (for statistics, see Table 1).

All participants tolerated the experimental procedures. As shown in Table 1, we found no significant difference between the three groups in age or measures of motor cortical excitability at baseline (Figure 4).

**Table 1:** Group values for age and stimulator intensities.

Experiment 1				
	iTBS-PPC + Grasp	iTBS-PPC + Rest	iTBS-Control + Grasp	Statistical comparison
Age (years)	24.0 ± 3.4	21.5 ± 2.1	24.5 ± 3.3	*F*(2,21) = 2.31 *p* = 0.12
AMT (% MSO) (MagPro MCF-B70 coil)	41.6 ± 8.0	41.7 ± 8.0	39.1 ± 6.7	*F*(2,19) = 0.29 *p* = 0.76
RMT (% MSO) (Magstim D50 B.I coil)	45.6 ± 8.6	46.9 ± 12.1	44 ± 9.3	*F*(2,21) = 0.16 *p* = 0.85
SI_1 mV_ (% MSO) (Magstim D70^2^ coil)	44.5 ± 6.7	47 ± 9.7	44.8 ± 9.7	*F*(2,21) = 0.19 *p* = 0.82

Experiment 2				
	iTBS-PPC + Grasp	iTBS-PPC + Rest	iTBS-Control + Grasp	Statistical comparison
Age (years)	26.4 ± 8.6	26.8 ± 8.3	27.8 ± 8.3	*F*(2,45) = 0.10 *p* = 0.90
AMT (% MSO) (MagPro MCF-B70 coil)	39.5 ± 5.4	37.9 ± 3.9	40.4 ± 6.2	*F*(2,40) = 0.82 *p* = 0.45
RMT (% MSO) (Magstim D50 B.I coil)	43.1 ± 5.6	44.5 ± 9.6	50 ± 10.9	*F*(2,45) = 2.67 *p* = 0.08
SI_1 mV_ (% MSO) (Magstim D70^2^ coil)	46.1 ± 7.3	47 ± 9.1	51.3 ± 11.7	*F*(2,45) = 1.36 *p* = 0.27

Data are presented as mean ± SD. Transcranial magnetic stimulation (TMS) intensity (expressed as a percentage of the maximum stimulator output, % of MSO) of active motor threshold (AMT) and resting motor threshold (RMT). SI_1 mV_ refers to the percentage of MSO required to produce a ~ 1 mV motor-evoked potential (MEP). Separate one-way analyses of variance (ANOVA) were used to confirm that the three groups did not differ in age or motor cortical excitability at baseline.

### Effects of the brain state during parietal stimulation on downstream motor cortical excitability

To test the hypothesis that manipulating the behavioral state during stimulation to PPC would affect motor plasticity associated with motor control, we compared changes in the excitability of the motor cortex by measuring the size of TMS-induced MEPs in the three stimulation groups.

#### Experiment 1

**Figure 5:**
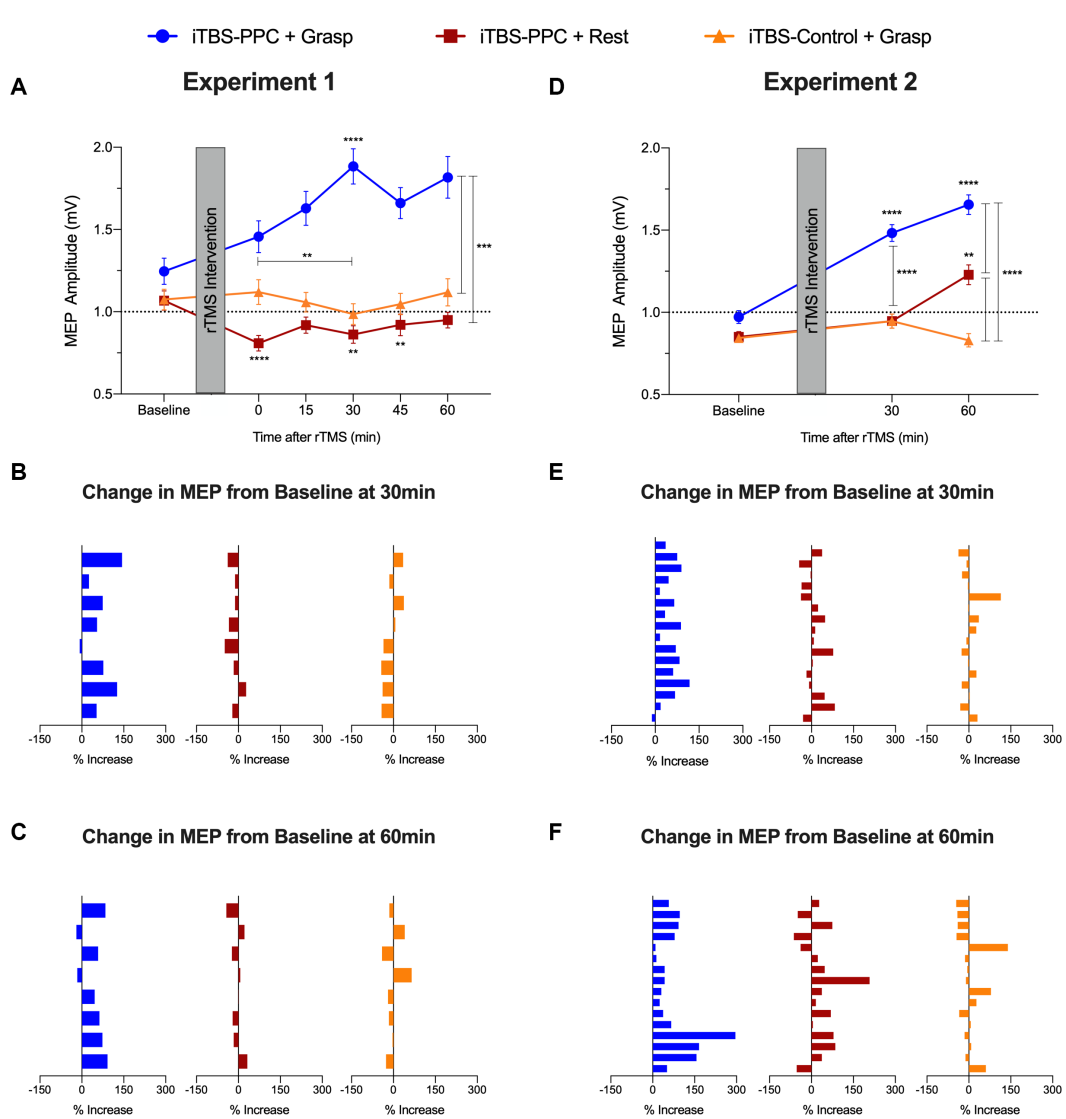
Group averaged motor evoked potential (MEP) amplitude (mV) for **(A)** Experiment 1 and **(D)** Experiment 2. Percentage change from baseline of MEP amplitude for each participant **(B)** 30 min and **(C)** 60 min post-stimulation in Experiment 1. Percentage change from baseline of MEP amplitude for each participant **(E)** 30 min and **(F)** 60 min post-stimulation in Experiment 2. Error bars denote the standard error of the mean (SEM). Asterisks indicate significant *post hoc* comparisons, *p ≤ 0.05, **p ≤ 0.01, ***p ≤ 0.001, ****p ≤ 0.0001.

There were significant main effects of the Intervention group (*F*_2,21_ = 4.17, *p* = 0.029, *η*_p_^2^ = 0. 28) and Time (*F*_5,3192.2_ = 3.33, *p* = 0.005, *η*_p_^2^ = 0.005) and a significant Time × Intervention group interaction (*F*_10,3192.2_ = 5.66, *p* < 0.001, *η*_p_^2^ = 0.02) on the MEP amplitudes; Figure 5A). *Post hoc* analyses revealed that MEP amplitudes for the iTBS_PPC + Grasp_ group were significantly different from baseline MEPs at 30 min (*p* < 0.0001), and the difference in amplitudes from immediate post-stimulation to 30 min was also significant (*p* = 0.002). For the iTBS_PPC + Rest_ group, MEP amplitudes were significantly different from baseline immediately after stimulation (*p* < 0.0001), at 30 min (*p* = 0.03), and 45 min (*p* = 0.03). Further *post hoc* analyses demonstrated that there were significant differences in MEP amplitude for the iTBS_PPC + Grasp_ group at every time point following baseline compared to both the iTBS_PPC + Rest_ and iTBS_Control + Grasp_ groups (all comparisons *p* < 0.0001, except between iTBS_PPC + Grasp_ and iTBS_Control + Grasp_ immediately post-stimulation, where *p* < 0.001. There were no significant differences in MEP amplitudes between iTBS_PPC + Rest_ and iTBS_Control + Grasp_ at any time point.

Closer inspection of the individualized normalized data showed highly consistent increases in the percentage change of MEP amplitudes across participants at 30 min and 60 min post-stimulation for the iTBS_PPC + Grasp_ group (MEP change (%) increased for 7 out of 8 participants at 30 min; sign test *p* = 0.07; MEP change (%) increased for 6 out of 8 participants at 60 min; sign test *p* = 0.29; Figures 5B,C). Conversely, neither iTBS_PPC + Rest_ (MEP change (%) increased for 1 out of 8 participants at 30 min; sign test *p* = 0.07; MEP change (%) increased for 3 out of 8 participants at 60 min; sign test *p* = 0.73) nor iTBS_Control + Grasp_ (MEP change (%) increased for 2 out of 8 participants at both 30 min and 60 min; sign test *p* = 0.29) affected the magnitude of change in the MEP at these time points.

#### Experiment 2

In line with the pilot experiment’s findings, Experiment 2 revealed significant main effects of the Intervention group (*F*_2,44_ = 5.64, *p* = 0.007, *η*_p_^2^ = 0. 2) and Time (*F*_2,3188.3_ = 26.91, *p* < 0.0001, *η*_p_^2^ = 0.02) and a significant Time × Intervention group interaction (*F*_4,3188.3_ = 22.95, *p* < 0.0001, *η*_p_^2^ = 0.03) on MEP amplitudes (Figure 5D). *Post hoc* tests indicated significant differences in MEP amplitudes for the iTBS_PPC + Grasp_ group from baseline at 30 and 60 min post-stimulation (*p* < 0.0001). MEP amplitudes for iTBS_PPC + Rest_ significantly differed between baseline and 60 min post-stimulation (*p* = 0.002) and between 30 and 60 min post-stimulation (*p* = 0.03), but not between baseline and 30 min post-stimulation. Conversely, no significant differences in MEP amplitudes were observed between any time points for the iTBS_Control + Grasp_ group. Further *post hoc* tests revealed no significant differences in MEP amplitudes among groups at baseline. However, at both 30 and 60 min post-stimulation, the iTBS_PPC + Grasp_ group showed significantly different MEP amplitudes compared to both iTBS_PPC + Rest_ and iTBS_Control + Grasp_ (*p* < 0.0001). At 60 min post-stimulation, the MEP amplitudes between the iTBS_PPC + Rest_ and iTBS_Control + Grasp_ groups also were significantly different (*p* < 0.0001).

The individualized normalized data showed consistent increases in the percentage change of MEP amplitudes from baseline across participants at 30 min and 60 min post-stimulation for the iTBS_PPC + Grasp_ group (MEP change (%) increased for 15 out of 16 participants at 30 min; sign test *p* < 0.0005; MEP change (%) increased for 16 out of 16 participants at 60 min; sign test *p* < 0.0001; Figures 5E,F). Conversely, neither iTBS_PPC + Rest_ (MEP change (%) increased for 10 out of 16 participants at 30 min; sign test *p* = 0.45; MEP change (%) increased for 12 out of 16 participants at 60 min; sign test *p* = 0.08) nor iTBS_Control + Grasp_ (MEP change (%) increased for 6 out of 16 participants at 30 min; sign test *p* = 0.45; and MEP change (%) increased for 7 out of 16 participants at 60 min; sign test *p* = 0.8) affected the magnitude of change in the MEP at these time points.

Together, these results indicate that the influence of PPC stimulation on motor cortical excitability depended on both the behavioral task being performed and the time at which the assessment was administered. Furthermore, this result reinforces that increased motor cortical excitability resulted from stimulation targeting a specific parietal-motor pathway. Critically, it is apparent that inducing functional activation in the cortical grasping network through a causal behavioral manipulation during parietal stimulation reliably alters downstream motor plasticity.

### The effects of brain state during parietal stimulation on motor performance

#### Experiment 1

**Figure 6:**
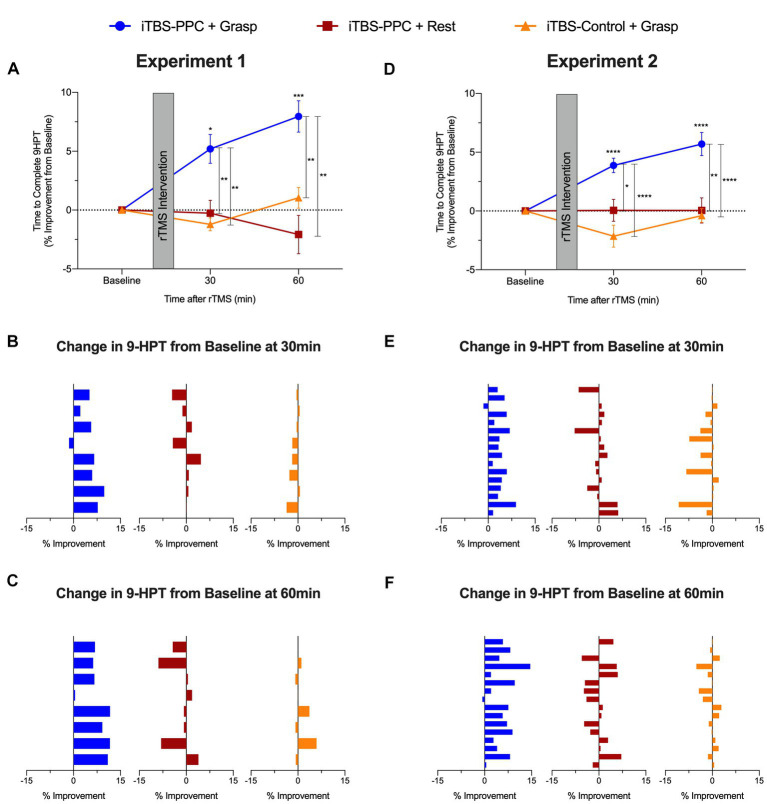
Group averaged percentage change from baseline to complete the nine-hole pegboard manual dexterity test (9-HPT) for **(A)** Experiment 1 and (**D**) Experiment 2. Positive values indicate a performance improvement. Mean percentage change from baseline to complete 9-HPT for each participant **(B)** 30 min and **(C)** 60 min post-stimulation in Experiment 1. Mean percentage change from baseline to complete 9-HPT for each participant **(E)** 30 min and **(F)** 60 min post-stimulation in Experiment 2. Error bars denote the standard error of the mean (SEM). Asterisks indicate significant *post hoc* comparisons, *p ≤ 0.05, **p ≤ 0.01, ***p ≤ 0.001, ****p ≤ 0.0001.

We examined the participants’ motor performance on a pegboard test after (30 and 60 min) each rTMS intervention. There was a significant main effect of the Intervention group (*F*_2,21_ = 11.54, *p* < 0.001, *η*_p_^2^ = 0.52), no main effect of Time (*F*_2,42_ = 1.68, *p* = 0.20, *η*_p_^2^ = 0.07), and a significant interaction between Time and Intervention group (*F*_4,42_ = 7.31, *p* < 0.001, *η*_p_^2^ = 0.41), on the symmetric percentage change from baseline of mean time to complete the pegboard task (Figure 6A). *Post hoc* analyses showed that motor performance significantly improved at each time of measurement for the iTBS_PPC + Grasp_ group compared to the TBS_Control + Grasp_ (30 min, *p* = 0.004; 60 min: *p* = 0.002) and at the 60 min for the iTBS_PPC + Rest_ group (*p* = 0.003). The time to complete the pegboard task decreased for the iTBS_PPC + Grasp_ group, as shown by *post hoc* analyses indicating a significant symmetric percentage change from baseline in motor performance at 30 min (*p* = 0.03) and 60 min (*p* < 0.001). Notably, there was no significant difference in the symmetric percentage from baseline in motor performance at each time point for the iTBS_PPC + Rest_ or the iTBS_Control + Grasp_ groups (all comparisons *p* ≥ 0.15).

Improvements in the percentage change from baseline in the time taken to complete the pegboard task were highly consistent across participants in the iTBS_PPC + Grasp_ group (7 out of 8 participants’ motor performance improved at 30 min; sign test *p* = 0.07; 8 out of 8 participants motor performance improved at 60 min; sign test *p* = 0.008) but not in the iTBS_PPC + Rest_ group (4 out of 8 participants motor performance improved at 30 min; sign test *p* = 1.27; 3 out of 8 participants motor performance improved at 60 min; sign test *p* = 0.73) or the iTBS_Control + Grasp_ group (2 out of 8 participants motor performance improved at 30 min; sign test *p* = 0.29; 3 out of 8 participants motor performance improved at 60 min; sign test *p* = 0.73; Figures 6B,C).

#### Experiment 2

The improvements in manual dexterity observed in Experiment 1 were replicated in Experiment 2. We found significant main effects of both Intervention group (*F*_2,45_ = 20.98, *p* < 0.001, *η*_p_^2^ = 0.48) and Time (*F*_2,90_ = 3.87, *p* = 0.02, *η*_p_^2^ = 0.08), as well as a significant Time × Intervention group interaction (*F*_4,90_ = 7.61, *p* < 0.001, *η*_p_^2^ = 0.25) on the symmetric percentage change in the time to complete the pegboard task from baseline (Figure 6D). *Post hoc* tests confirmed that the iTBS_PPC + Grasp_ group showed significant differences from the other intervention groups, and their performance improved over time. Specifically, the iTBS_PPC + Grasp_ group showed significantly different symmetric percentage changes in performance at both 30 and 60 min post-stimulation when compared to either of the other groups (iTBS_PPC + Rest_, 30 min: *p* = 0.01, 60 min: *p* = 0.002; iTBS_Control + Grasp.,_ 30 min: *p* < 0.001; 60 min: *p* < 0.001). Similar to the findings in Experiment 1, only the iTBS_PPC + Grasp_ group displayed improvements in performance over time, with their symmetric percentage change in time to complete the pegboard task being significantly different from baseline at both 30 and 60 min (*p* < 0.001). In contrast, neither of the other groups showed significant differences at either time (all comparisons *p* > 0.07).

Improvements in the percentage change from baseline in the time taken to complete the pegboard task were highly consistent across participants in the iTBS_PPC + Grasp_ group (15 out of 16 participants’ motor performance improved at both 30 and 60 min; sign test *p* < 0.0005) but not in the iTBS_PPC + Rest_ group (10 out of 16 participants motor performance improved at 30 min; sign test *p* = 0.45; 8 out of 16 participants motor performance improved at 60 min; sign test *p* = 1.2) or the iTBS_Control + Grasp_ group (5 out of 16 participants motor performance improved at 30 min; sign test *p* = 0.21; 7 out of 16 participants motor performance improved at 60 min; sign test *p* = 0.8; Figures 6E,F). Thus, motor improvement occurred reliably only when the functional state of the grasp network was engaged with a motor task during the administration of parietal stimulation, not for either control condition. These results underscore the consistent benefits of state-dependent parietal stimulation on manual dexterity across both experiments.

**Figure 7:**
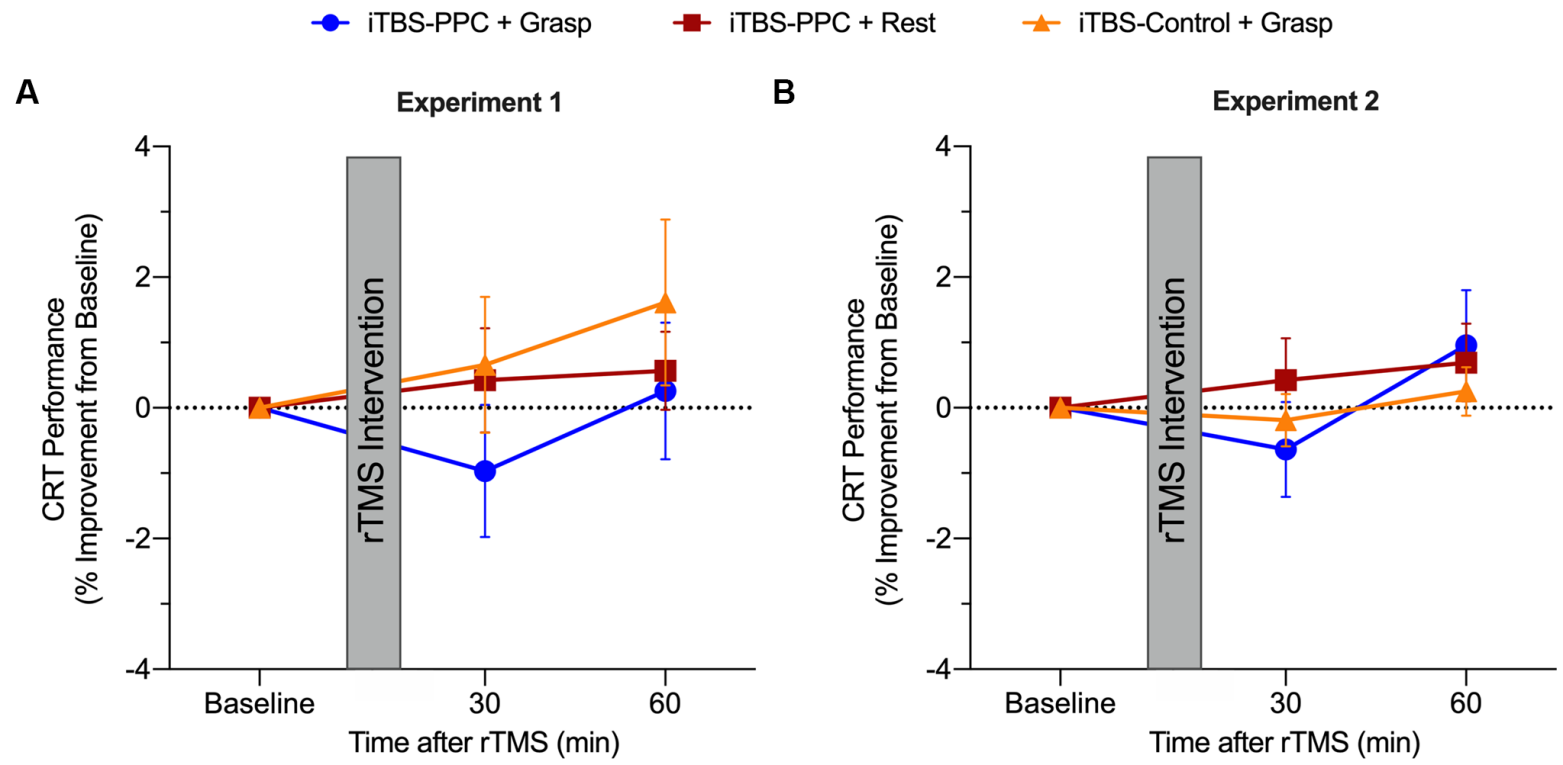
Group averaged percentage change from baseline for the choice-reaction visuomotor task (CRT) for 30 and 60 min post-stimulation for **(A)** Experiment 1 and **(B)** Experiment 2. Positive values indicate a performance improvement. Error bars denote the standard error of the mean (SEM).

To assess the specificity of the effects of stimulation on motor performance, we compared reaction times for a control visuomotor CRT task that does not involve dexterous hand shaping and object manipulation in both experiments. Across both experiments, no significant differences in visuomotor performance were found across intervention groups or time. In the pilot experiment, after fitting and testing a linear mixed-effect model, there were no main effects of Time (*F*_2,42_ = 1.42, *p* = 0.25, *η*_p_^2^ = 0.06) or Intervention group (*F*_2,21_ = 0.03, *p* = 0.97, *η*_p_^2^ < 0.001), and no significant interaction (*F*_4,42_ = 0.60, *p* = 0.67, *η*_p_^2^ = 0.05; Figure 7A). Similarly, in the second experiment (Figure 7B), there was no significant main effect of Time (*F*_2,90_ = 2.64, *p* = 0.07, *η*_p_^2^ = 0.06), Intervention group (*F*_2,45_ = 1.60, *p* = 0.21, *η*_p_^2^ = 0.07), or interaction (*F*_4,90_ = 0.81, *p* = 0.52, *η*_p_^2^ = 0.03). These findings are consistent with the hypothesis that the effect of parietal stimulation on motor performance is specific to planning and execution states for object-directed grasps rather than a general attention or performance benefit. Motor improvement was selective for skilled object-directed grasps and occurred reliably only when the targeted cortical motor planning network was engaged with a motor behavior at the time of parietal stimulation.

## Discussion

The current study describes brain and behavior responses to intermittent theta burst stimulation to PPC applied during two distinct endogenous states of neural activity in the motor system (i.e., brain state at rest versus during action planning and execution). Delivering iTBS to PPC when the cortical grasp network is engaged with a motor task increases the downstream excitability of an interconnected M1 region responsible for fine-motor action and concomitantly improves skilled motor performance for up to an hour. These findings demonstrate that the effects of parietal network-targeted stimulation are brain-state dependent and can influence motor plasticity beyond the stimulated region with high specificity to improve skilled motor control of hand actions immediately after stimulation.

It has been commonly found that there is a large degree of variability in brain activity and behavioral responses following rTMS (Ridding and Rothwell, 2007; Nicolo et al., 2015; Ziemann and Siebner, 2015; Corp et al., 2020; Ozdemir et al., 2020, 2021). For motor control, most work has examined the neural effects of rTMS on a brain at rest (Siebner et al., 2009a; Bergmann et al., 2016). However, recent work has proposed that this variability can be partially explained by state-dependent effects, in which the stimulation response depends on the ongoing level of brain activity during stimulation (Silvanto et al., 2008; Pasley et al., 2009; Romei et al., 2016; Bergmann, 2018; Bradley et al., 2022). In our study, we show that the direction of change in excitability is influenced by the physiological state of the targeted parietal-motor grasp network during stimulation. Our results suggest that PPC’s facilitatory influence during grasping may cause recurrent excitation, leading to long-term potentiation-like changes in cortical excitability when stimulated. In contrast, stimulating during periods of PPC-mediated inhibition, such as during rest, may reduce neural activation, resulting in less potentiation. This explanation aligns with studies indicating that the ongoing activity level at the time of stimulation influences corticospinal excitability (Bestmann et al., 2008a; Bestmann and Krakauer, 2015; Zrenner et al., 2018; Naros et al., 2019; Schaworonkow et al., 2019) and is consistent with recent cortico-cortical TMS findings showing remote excitability effects of the parietal cortex on motor cortex reverse in direction with the motor state (Koch et al., 2008; Vesia et al., 2013, 2017).

The current findings also align with TMS’s neural and behavioral effects when manipulating sensory, attentional, and cognitive states. Recent findings also have demonstrated that co-administration of low frequency rTMS to the motor cortex with motor training can enhance motor plasticity and improve motor skills in both damaged and intact brains (Bütefisch et al., 2004; Thabit et al., 2010; Buetefisch et al., 2011, 2015; Revill et al., 2020). In addition, cognitive manipulations that direct attention to the hand during rTMS have been shown to produce larger increases in motor cortical excitability (Stefan, 2004; Conte et al., 2007). Other approaches have been used to modulate brain excitability before TMS by selectively preconditioning a specific neuronal population using either stimulation (Siebner, 2004; Ni et al., 2014; Opie et al., 2019) or behavioral adaptation (Silvanto et al., 2007) protocols. For instance, perceptual adaptation has been shown to augment the TMS-induced neural representation of observed motor behavior (Silvanto and Cattaneo, 2014). Our current results extend these prior findings on state-dependent motor responses to rTMS to provide novel physiological evidence that engaging the parietal–frontal network for goal-directed hand movements during parietal stimulation can affect cortical motor output for up to an hour.

The state dependency of neuronal responses to rTMS also can be found in interconnected brain areas (Siebner et al., 2009b). Indeed, it is well established that the effects of stimulation propagate beyond the stimulation site to impact functionally specific brain networks (Siebner et al., 2009a; Fox et al., 2012; Beynel et al., 2020; Lynch et al., 2022). Importantly, the effects of stimulation on brain networks can be influenced by the activation state of interconnected regions within the functional network (Ruff et al., 2006, 2008; Blankenburg et al., 2008, 2010; Moisa et al., 2012). It is possible that the ongoing activity and inherent excitability of neurons can influence the spread of neural excitation within the targeted area and to other regions in the brain. As a result, synchronizing neural firing patterns with stimulation can strengthen connections between neurons and facilitate state-specific changes in the brain (Siebner et al., 2022). For example, activating the motor system with a behavioral task, such as the performance of an isometric hand grip during premotor cortex stimulation, influences contralateral cortex activity (Bestmann et al., 2008b). Cortico-cortical interactions that can be probed with two TMS coils over two connected brain areas have shown dynamic changes in excitability when individuals plan actions (Koch and Rothwell, 2009; Lafleur et al., 2016; Hallett et al., 2017; Goldenkoff et al., 2020; Malderen et al., 2022). Furthermore, our previous dsTMS experiments show that PPC regions involved in encoding hand movements exert an inhibitory influence on motor output at rest. Interestingly, this net inhibitory drive at rest in PPC is facilitated during the preparation of a grasping movement (Vesia et al., 2013, 2017). We, therefore, reasoned that capitalizing on the physiological state of the brain using multi-focal TMS methods can selectively target active neurons when delivering stimulation to the parietal location to enhance the specificity of excitation to the connected motor regions. This approach may increase the excitability of specific neural pathways associated with movement by modulating the connections between pre- and post-synaptic neuronal activities through Hebbian mechanisms (Hebb, 1949; Markram et al., 2011), inducing LTP-like changes in synaptic strength (Suppa et al., 2016). In the current study, intermittent theta burst stimulation may have induced long-lasting changes in cortical excitability by modulating calcium influx via the post-synaptic membrane, resulting in LTP-like effects on cortical synapses (Huang et al., 2011; Suppa et al., 2016). The underlying mechanisms of the brain-state-dependent TMS effects on motor function observed in our data may be activity-dependent plasticity, whereby control over the cortical state with a voluntary movement during stimulation boosts the response in the activated brain network (Siebner et al., 2009b); *cf.* (Paulus and Rothwell, 2016). The current results add to prior research demonstrating that theta burst stimulation to premotor (Huang et al., 2018) and PPC (Premji et al., 2011) regions can impact downstream cortical motor plasticity. Altogether, these findings demonstrate that the effect of stimulation on downstream motor cortical excitability depends on the current state of excitation of the connected brain region being stimulated within the functional network.

The present findings also demonstrate that this state-dependent modulatory effect can improve behavior immediately after stimulation. For example, we found that theta burst stimulation to the grasping network selectively improves skilled motor performance when the network is active relative to when it is at rest. This result is consistent with previous work in the visual domain, indicating robust state-dependent effects when pairing stimulation with neural activity functionally tuned to visual motion stimuli (Chiappini et al., 2018). One possible mechanism of the immediate state-dependent effect is that selective neural representations and pathways underlying the perceptual and behavioral processes are more susceptible to stimulation (Silvanto et al., 2008; Pasley et al., 2009; Romei et al., 2016; Bradley et al., 2022). Future neuroimaging work is necessary to characterize the neural basis of this neural response, particularly at mesoscale brain circuits that subserve voluntary motor control.

The convergence of the current neurophysiological and behavioral findings strongly suggests that variability in neural activity levels at the time of stimulation contributes to the variability of the responses to rTMS in the motor system. In the current study, the group-averaged data showed that applying theta burst stimulation during a constrained high-activity state through a causal behavioral manipulation improves motor function immediately after stimulation rather than using the same pulse train during spontaneous neural activity discharge while participants are at rest. A closer inspection of individual results clearly shows two distinct patterns diverging based on the functional context of neural activity during stimulation. The group that received brain-state-dependent parietal stimulation showed significant and consistent increases in both excitability and performance in all participants. In contrast, the effects of stimulation on motor plasticity and motor performance changes varied in magnitude and direction across individuals within the control conditions. This implies that variations in resting-state brain activity may influence individual differences in TMS responses and can be reduced by task manipulations (Silvanto et al., 2008; Silvanto and Pascual-Leone, 2008; Siebner et al., 2009b, 2022; Romei et al., 2016; Bergmann, 2018). This relationship may explain, in part, the considerable individual variation in brain and behavior responses within healthy and patient population studies reported in brain stimulation research. Such state-dependent TMS methods can identify novel neural paths to modify the output of the motor cortex and possibly translate into therapeutic approaches that underpin hand control for neurological disorders with aberrant plasticity. Notably, motor impairments after stroke often can be explained by abnormalities in parietal–frontal circuits subserving the integration of sensory input with motor commands, thus demonstrating network-level dysfunction of neural interactions for sensorimotor control (Grefkes and Fink, 2011; Guggisberg et al., 2019). Most therapeutic stimulation has focused on frontal motor circuits (Morishita and Hummel, 2017), encompassing the primary motor cortices, premotor cortices, and supplementary motor areas. Here, we focused on interactions between PPC, a higher-order area significantly involved in action-related processes, and frontal motor areas (Andersen and Cui, 2009). Even though these network effects are relevant to therapeutic response (Fox et al., 2014; Horn and Fox, 2020), few studies have focused modulatory stimulation on the PPC component, an important ‘brain hub’ (Grefkes and Fink, 2011, 2014; Grefkes and Ward, 2014) of a well-characterized parietal–frontal grasping network (Grafton, 2010; Davare et al., 2011; Vesia and Crawford, 2012; Turella and Lingnau, 2014). This is important because higher levels of functional connectivity in parietal–frontal circuits in the motor system have been related to more favorable motor outcomes after stroke (Schulz et al., 2015, 2016). Given that the current results provide evidence for parietal contributions to motor function, we propose that targeting higher motor areas such as PPC with rTMS, primarily when functionally engaged with other interconnected frontal cortical regions, might be a better alternative for stroke patients with greater sensorimotor impairments (Plow et al., 2014, 2016). Further research is needed to determine the relevance of the proposed rTMS approach in clinical settings.

The current study has some limitations worth noting. First, the number of female participants in Experiment 2 was greater than males. Recent work has highlighted the influence of sex on the brain and behavior responses to TMS (Hanlon and McCalley, 2022). This may relate to various biological metrics, such as the distance between the scalp and cortex, gray matter density, and estradiol and progesterone levels. Still, sex is unlikely to account for the current results because it would be counterbalanced across the intervention groups. In addition, we personalized stimulation intensity based on each participant’s neuroanatomy to minimize variance in cortical target site intensities (Stokes et al., 2005). It is also important to consider that the current study did not implement a sham control. We, therefore, cannot rule out the TMS-induced placebo effects on brain and behavioral outcomes (Boucher et al., 2021). We would expect, however, variance in brain and behavior responses in all intervention groups. Yet, our results showed clear and consistent effects of brain-state-dependent parietal TMS on motor excitability and manual dexterity, with notable differences between intervention groups. Future research could benefit from including sham TMS to better differentiate the effects of time on motor function.

In summary, our findings demonstrate that brain-state-dependent stimulation of a higher-order node in the cortical grasping network can alter motor cortical excitability beyond the stimulation site, leading to improved motor control of hand movements for up to an hour. Whether these changes in brain and behavior persist beyond the one-hour period we tested remains to be seen. Similarly, because multiple consecutive days of stimulation can produce long-lasting cumulative effects (Wang and Voss, 2015; Freedberg et al., 2019a,b), future studies should investigate the duration and magnitude of state-dependent changes on motor function caused by multiple-day stimulation, which could be particularly relevant to a clinical cohort. As our data indicate, rTMS results could be more consistent by controlling the behavioral state at the time of stimulation to induce network-specific plasticity in the motor system. It may prove useful to employ this methodological approach to optimize targeted neuromodulation strategies with practiced movements for treating a wide range of neurological disorders marked by movement dysfunction, such as stroke and Parkinson’s disease.

## Data availability statement

The raw data supporting the conclusions of this article will be made available by the authors, without undue reservation.

## Ethics statement

The studies involving humans were approved by the University of Michigan IRB. The studies were conducted in accordance with the local legislation and institutional requirements. The participants provided their written informed consent to participate in this study.

## Author contributions

EG: conceptualization, methodology, formal analysis, writing – original draft, writing – review and editing, visualization. JD: formal analysis, writing – review and editing. DD: investigation, methodology, formal analysis, writing – original draft, writing – review and editing, visualization, project administration. TL: methodology, writing – review and editing, supervision. KM: investigation, methodology, formal analysis, project administration, writing – review and editing. JB: methodology, formal analysis, writing – review and editing. ST: methodology, writing – review and editing. TP: methodology, writing – review and editing. MV: conceptualization, investigation, methodology, resources, writing – review and editing, supervision, funding acquisition. All authors contributed to the article and approved the submitted version.

## Footnotes

^1^www.nihtoolbox.org
